# The Constructive Overlap: A Study of Multiplex Ties in Students’ Study-Related Networks and Academic Performance

**DOI:** 10.1007/s10755-021-09576-4

**Published:** 2021-09-09

**Authors:** Annika Fjelkner-Pihl

**Affiliations:** Center for Higher Education Development, Kristianstad University, Kristianstad, Sweden

**Keywords:** Social network analysis, Academic outcome, Higher education, Commuter student, Multiplex relations

## Abstract

This article adds to a growing body of literature on how various types of social relations can work synergistically to promote students' academic success. Students’ study-related social networks affect academic outcome in higher education. The network literature in education generally explores students’ various relations separately, rather than their multiplex relations or when *individuals share several relations*. This approach risks missing the full complexity of the student experience. The aim of the present study is to add to the discussion on student social networks and attainment in higher education by further exploring multiplex relations maintained in a specific study program, in which a large share of students in the cohort commute. A survey was distributed to students in one cohort (*n* = 146). The findings revealed that, in this cohort, students’ friendship, working and learning networks overlap substantially, and that centrality in the friendship and in the student multiplex networks was positively and significantly related to academic outcome, whereas centrality in the working and learning networks was not. Points for future research are suggested, and practical implications for those supporting student learning in higher education are discussed.

## Introduction

The classroom is a central place for the formation of supportive relations, and university students generally perform better in relationship-rich environments (Felten & Lambert, 2020). The transition to online education during the Covid-19 pandemic has made the importance of social relations even more apparent. Students report a decline in well-being as interaction between students has declined and co-studying networks have become sparser (Elmer et al., 2020).

It seems as though the social control and peer pressure existing within student social networks motivate them to succeed (Eggens et al., 2007). As the student body becomes more diverse, and more students live at home and commute to university rather than living on or close to campus (e.g., London Higher, 2019; Pokorny et al., 2017; Thomas, 2019), it may be difficult for them to create and maintain supportive networks. Important work is done on different levels—with faculty, staff and students—to enhance the student experience (Felten & Lambert, 2020). The focus of the present study is on how the students themselves form relations in and around the classroom.

Several studies have explored student friendship and learning relations and their effect on academic outcome (e.g., Eggens et al., 2007; Hommes et al., 2012; Rienties & Tempelaar, 2018). Centrality in a social network has been found to be positively linked to academic success, even though social network relations had a greater effect on student learning than prior performance. At the same time, an independent analysis of student relations may be oversimplistic. For this reason, there is a need to acknowledge that several relations largely overlap and that it may be these so-called multiplex relations that positively affect learning and academic outcome. Multiplex relations occur when individuals share several relations, for example, if you have a friend who is also your co-student and your cousin (e.g., Kuwabara et al., 2010; McCabe, 2016). In addition, studies have shown that overlapping (multiplex) study-related relations are more likely to remain after college (McCabe, 2016) and have also been more resilient during the Covid-19 pandemic (Elmer et al., 2020).

However, studies focusing on multiplex relations in higher education are rare. Network studies have instead focused largely on how different types of relations in isolation contribute to academic outcome. The aim of the present study is to explore how students form working, learning and friendship relations and to what extent these overlap in multiplex relations as well as to look at how uniplex and multiplex relations relate to academic outcome. Thus, the present study adds a new approach by shifting from the study of independent student friendship, learning and working relations to a more complex understanding of student relations and academic achievement, and possible implications for practice.

## Literature Review

First, there is a need to acknowledge previous research on factors influencing academic outcome. These are partially personal background factors that are difficult for universities to influence. For example, research has consistently found that IQ and previous performance are the strongest predictors of academic outcome (e.g., Cambell & Dickson, 1996; Rosander & Bäckström, 2014). Other factors, for example, engagement (e.g., Kahu, 2013; Kuh, 2009; Thomas, 2012; Trowler & Trowler, 2010 and social network formation (e.g. Biancani & McFarland, 2013; Eggens, et al., 2007; McCabe, 2016) may be influenced by universities, because these factors are related to students’ experiences. However, the picture is complex, and several factors are intercorrelated. The remaining part of the literature review will discuss how student social networks, or study-related relations, affect academic outcome.

### Student Friendship, Working and Learning Relations and Academic Outcome

Social network analysis (SNA) offers a structured way to explore student networks, focusing on the relations between actors (Hollstein, 2014). Social networks can be defined as the study of linkages or ties (relations) between a specific set of social actors (nodes) in, for example, a workplace or a family. An actor’s importance in a network is generally conceptualized as the actor’s centrality within a network, for instance, the number of relations a student has within a specific network (e.g., Grunspan et al., 2014). There is consistent evidence showing that students form homophilic networks, that is, they prefer friends like themselves regarding race/ethnicity, socioeconomic background, age, and cultural preferences. This tendency may be due to either preference or opportunity. Initial group division has been found to be a strong predictor of relations. In modules where students worked together on well-aligned assignments and tasks, students developed cross-cultural friendship relations, at least temporarily (Rienties & Nolan, 2014).

Relations are operationalized differently in different studies, but most studies distinguish between instrumental, work-related relations and affective, friendship relations (Rienties & Tempelaar, 2018). Friendship networks are expressive networks based on trust and involve passive information diffusion (Hommes et al., 2012, p. 747); they are normally considered to involve what Granovetter (1973) defined as strong ties (McCabe, 2016). School-related working and learning networks, on the other hand, provide instrumental support (Chen et al., 2012), such as sharing of notes or solving an assignment together, and as such are often considered to involve weak ties. The number of relations a student maintains varies, but one consistent pattern is that students seem to have fewer learning relations than friendship relations (e.gRienties et al., 2013; Shah et al., 2017).

Centrality in a social network is positively linked to academic success, as measured by GPA (e.g., Grunspan et al., 2014). For example, Hommes et al. (2012) studied the influence of social networks on academic outcome in a Dutch medical school (*n* = 301). They found that social networks were associated with students’ social integration, and that social network relations had a greater effect on student learning than did prior performance. Prior performance was found to influence students’ centrality in the network, with the high performers being in central positions and the students with the lowest performance being in the periphery (Hommes et al., 2012). In another study, Tómas-Miquel et al. (2016) found a positive relationship between centrality in the academic (work) network and academic success, and a negative relationship between centrality in the friendship network and academic success. Hence, students’ social network has been measured in various ways, and there are inconclusive results on which type of relation matters most for academic success (Rienties & Tempelaar, 2018; Tómas-Miquel et al., 2016).

There are two important aspects to consider. First, studies often focus on specific modules and groups constructed by teachers, either as teams in specific modules or learning communities. This means that the inter-group learning relations may be the relations students normally work/learn with. In a cohort of students who take almost all courses together, this could mean that students’ *intra-group* working/learning relations are what Rienties and Tempelaar (2018) define as *inter-group* working/learning relations. Second, the types of relations or networks studied have been defined somewhat differently in different studies. In some studies, researchers move between discussing the contribution of students’ overall social network to discussing a specific type of relation (e.g., working or learning relation), as if they were identical. This means that it is difficult to determine the contribution of each type of relation or what type of relation is being discussed.

In the present study, friendship networks are defined as peers with whom students socialize in and around the classroom. These are expressive relations, but may include passive information diffusion (Hommes et al., 2012). Academic, working and learning networks include individuals with whom students communicate formally or informally about study-related topics. Thus, the main difference between the present study and previous studies is that students were not assigned to any specific groups at the time of the study. Instead, they reported on whom they considered they had learned from or worked a lot with during the past 2 years of study, rather than just during one module. Based on the above discussion, it is possible to suggest that both students’ social (affective) friendship relations and instrumental working and learning relations contribute to academic outcome. Multiplex relations are when students share all three relations. The types of network relations discussed in the present paper and the type of support they offer are summarized in Table 1.

**Table 1:** Types of Network Relations and Support Offered

Type of relation	Definition	Type of support
Friendship	People in class students socialize with in and around the classroom	Affective
Learning	People in class students have learned from during their studies	Instrumental
Working	People in class students have frequently cooperated with during their studies	Instrumental
Multiplex	People in class with whom students share all three relations (friendship/learning/working)	Affective & instrumental

### Multiplexity in Students’ School-related Networks

Many relations are multiplex, but relationships in education are still often treated as separate, independent constructs (uniplex), which may be misleading. There are few studies on multiplex relations in higher education, but there are organizational studies exploring individual performance benefits of multiplex relationships (Shah et al., 2017), tradeoffs of multiplex relationships and association with job performance (Methot et al., 2016), or on the role of tie content in the evolution of multiplex relations in interorganizational networks (Ferriani & Fonti, 2013). Multiplex relations can be assumed to be particularly strong when affective ties overlap with instrumental ties. Thus, multiplex relations that combine friendship, learning and working relations could be defined as strong ties, which are more closely linked to emotion, identity (Granovetter, 1973) and change in values and behavior (Centola, 2018).

The relation between multiplex relations and performance in an educational setting is underexplored. It is often presumed that social exchange takes place in a similar manner between all types of relationships in a network, although goodwill (social capital) exists to a different extent in relationships. This may mean that it can be theoretically problematic to treat learning, working and friendship relations as though they were identical, as such an approach may lead to erroneous conclusions (Shah et al., 2017). It is not possible to determine whether the effects of a friendship network can be solely attributed to the friendship itself, or whether that friendship is interwoven with a work-related component where friends also share instrumental support and information (Methot et al., 2016).

In some of the few studies in this area, Chen et al. (2012) described students’ multiplex relations in a Chinese MPA program, McCabe (2016) discussed how students develop academic and social relations, and multiplex relations over time in university in the US, and Shah et al. (2017) explored the effect of uniplex and multiplex relations on performance in teams of middle-manager MBA students. Friendship network density was not significantly related to group performance, although, in groups with strong friendships, constructive controversy boosted performance, in contrast to groups with weak friendship relations, where constructive controversy harmed performance. Hood et al. (2017) explored the effect of conflicts and multiplex relations on team performance in 120 teams of business students. They found that conflicts among team members who were also friends negatively impacted team performance, whereas conflicts between non-friends had a positive effect on team performance.

In sum, the substantial overlap between the different friendship, working and learning relations reported in previous research (e.g., Chen et al., 2012), multiplex relations (McCabe, 2016), and the substantial overlap found in the present data point to the relevance of exploring student multiplex and uniplex relations and academic outcome.

### Aim & Research Questions

The aim of the present study was to explore how students form working, learning and friendship relations and to what extent these overlap in multiplex relations, as well as to look at how uniplex and multiplex relations are related to academic outcome. Thus, the present study adds a new approach by shifting from the study of independent student friendship, learning and working relations to a more complex understanding of student relations and academic achievement:

- (RQ1) To what extent do students in a specific program develop relations with other students and what are the characteristics of the networks formed?
- (RQ2) What is the relation between students’ friendship, learning and academic work networks and academic outcome in this specific context?
- (RQ3) What is the relation between students’ multiplex relations and academic outcome in this specific context?

## Method

### Research Design

The analysis is based on data from an exploratory survey using self-reports. Quantitative relational data were collected in a paper survey (*N* = 109) at the end of the fourth term of study, where one term equals 20 weeks of study. The present article reports on a quantitative analysis of student uniplex and multiplex study-related relations and their effect on academic performance.

### Study Context

The present study focused on a cohort of business students (*N* = 146) at a teaching-intensive university in Sweden in the spring and fall semester of 2016. Few in this cohort lived on or close to campus, and about 70% commuted to school, the commute taking between 1 and 3 h a day. Thirty-seven percent had an immigrant language background, and only 30% had two parents with an academic degree.

Students were divided into three classes of between 40 and 70 students, which they followed for 3 years. This is in stark contrast to the situation for students in most reported studies on student social networks in university settings, where students had more flexibility to choose their courses and/or were more likely to live on campus and form networks within their living arrangements/dormitories or in organized learning communities.

The student population of the present study was less culturally diverse than the populations in studies focusing on ethnicity or student social networks and race. The Swedish National Agency for Higher Education (2018) advocates widening participation and recruitment based on gender, social background, immigrant background, and domicile (counties and municipalities). On average, the proportion of immigrant students was 24% in 2016/2017, which was representative of the population as a whole (SCB, 2021).

Students worked in assigned teams during their first semester, with a mix of students in terms of gender, language background (native/immigrant) and place of residence (commuter/local) to enable them to form study-related relations. Together, these teams solved different study-related tasks. In subsequent courses, students were mainly free to self-select their groups.

### Sample and Procedure

Individual-level background data were collected from 146 students (men: *n* = 64; women: *n* = 82) who were part of a cohort of students enrolled in a business administration program divided into three distinct specializations: accounting and auditing (Specialization A), bank and finance (B), and international business and marketing (C). They studied together the first year and to some extent in the second year.

*Relational network data* were collected from 106 students (men: *n* = 40; women: *n* = 56; response rate 73%) (Table 2). The network data were collected via a paper survey in class during a lecture in spring 2017. The completion time was about 30 min, and participation was voluntary.

**Table 2:** Sample characteristics

	A	B	C
N	73	34	39
No. female students No. native students	46 (63%) 46 (63%)	14 (41%) 22 (65%)	22 (56%) 24 (62%)
Average age (years) Average SweSAT** Previous GPA** Academic outcome*	25.8 (SD = 4.9) 0.87 (SD = 0.30) 17.27 (1.92) 144.10 (SD = 44.57)	24.7 (SD = 2.0) 0.96 (SD = 0.25) 16.66 (1.76) 151.10 (SD = 40.05)	24.3 (SD = 2.2) 0.79 (S = 0.29) 16.55 (2.26) 146.36 (SD = 42.58)
Av. commute ***	2.23 (1.43)	1.49 (1.21)	1.33 (1.31)

* Total credits achieved in three years; ** SweSAT (Swedish national scholastic aptitude test). Max = 2; National average = 0.9; *** Average time (h) spent commuting

Students were informed about the aim of the study and how the data would be used and presented. They were asked to give their written consent and were ensured confidentiality in the handling and presentation of data, in line with the university’s ethical guidelines.

### Measures

The instrument used in the first part of the study mapped the working, learning and friendship relations students maintained. It was assumed that because students were at the end of the second year, they had likely formed both friendship and academic work relations. Information concerning sociodemographic characteristics were obtained from secondary data (Fjelkner, 2020).

#### Networks

A closed network (e.g., Rienties, & Templaar, 2018; Tómas-Miquel et al., 2016), roster recollection method (Tómas-Miquel et al., 2016) was used, that is, students were asked to select the students they were friends with from a list of names of all the students registered in the given specialization. To explore student networks, participants received a list of all students enrolled in their specialization and were asked to mark students whom they “*work a lot with,*” “*have learned from,*” and “*are friends with.*” Regarding the question “I am friends with,” Swedish has two commonly used words for *friend*, one conveying the meaning of close friend (*vän*) and the other someone one is better acquainted with (*kompis*), but still more a friend than a mere acquaintance. The word finally used for friend was *kompis*, indicating that students should mark not only their closest friend/s in the group, but everyone with whom they regularly hang out, at least in class or during breaks.

#### Sociodemographic Predictors

Sociodemographic predictors included background questions regarding age, gender, upper secondary education, language background (native Swedish speaker/immigrant Swedish speaker), time spent commuting and parents’ academic background. The background data used in the present study were secondary data collected in the same cohort for an earlier study on student readiness for higher education studies (REF); that study showed that all of these factors affect academic outcome, in line with findings from previous research (e.g., Krause et al., 2005; Trowler & Trowler, 2010; Yorke, 2004). Students were asked to indicate the language background of their parents. Students with at least one native Swedish-speaking parent were classified as native, whereas students with two immigrant Swedish-speaking parents were classified as immigrant students.

Parents’ educational background has been used as a proxy for social background (Schmidt, 2012). Students indicated the highest degree obtained by their parents (compulsory, upper secondary, tertiary). Responses were then collapsed into a dummy variable (0 = upper secondary diploma or less; 1 = university degree). Students reported time spent commuting in the survey. To determine where students commuted from, information on place of residence was collected from the university administration’s system.

#### Academic Predictors

Grade point average (GPA) from upper secondary school and Swedish scholastic aptitude test scores (SweSAT) were used as academic predictors; both were retrieved from the university student administration system. Grade point average (GPA) was measured as the admission entry points, which is an average based on upper secondary school grades. SAT scores have been found to predict academic outcome, although the research is inconclusive (Kuncel et al., 2005; Lyrén et al., 2014). The use of standardized test scores has been criticized due to concerns about issues such as test fairness as well as the risk of built-in biases that might disfavor ethnic and cultural groups (Haughbrook, 2021). However, the predictor was still included, as one third of all Swedish students are admitted to university based on their SweSAT scores.

#### Analysis

Freeman’s in-degree centrality was used to measure centrality for the working/ learning/friendship networks (Grunspan et al., 2014). The in-degree centrality measures the number of incoming ties indicating how sought out or prominent an actor is in the network. The in-degree centrality was used to limit the bias inherent in self-reported networks ties (Hanneman & Riddle, 2005). Centrality measures were analyzed using the software UCINET v. 6, a program developed for social network analysis (Borgatti et al., 2002). Networks were visualized using Netdraw, in UCINET. To assess whether a pair of networks is structurally similar at a system level, a quadratic assignment procedure (QAP) was performed, correlating each pair of matrices (Hanneman & Riddle, 2005).

Whole networks were used to calculate the number of relationships students were involved in, that is, in-degree centrality. Work-focused centrality was measured as the count of the total number of students who indicated a work-related tie with a specific student. To explore RQ 3, two other measures were used: multiplex centrality and uniplex socially focused *centrality*. The multiplex networks were created using the multiplex routine in UCINET (Methot et al., 2016). A tie was considered multiplex only if an individual reported having all three relations with another student.

A bivariate correlations model was used to explore the relation between academic outcome and the various sociodemographic, academic, and network variables. Variables that correlated significantly with academic outcome were then used in subsequent analyses. Hierarchical regression models were used to analyze the effect of students’ position in the networks and background factors on academic outcome (dependent variable). Students with no ties were eliminated from the analysis. Differences were considered statistically significant if *p* < 0.05, two-tailed. SPSS (IBM Corp. Released 2016. IBM SPSS Statistics for Windows, Version 24 Armonk, NY: IBM Corp.) was used for all analyses. The control variable gender was included, in line with other studies in the field (e.g., Eggens et al., 2007; Tómas-Miquel et al., 2016).

## Results

### Descriptive Statistics

**Fig. 1:**
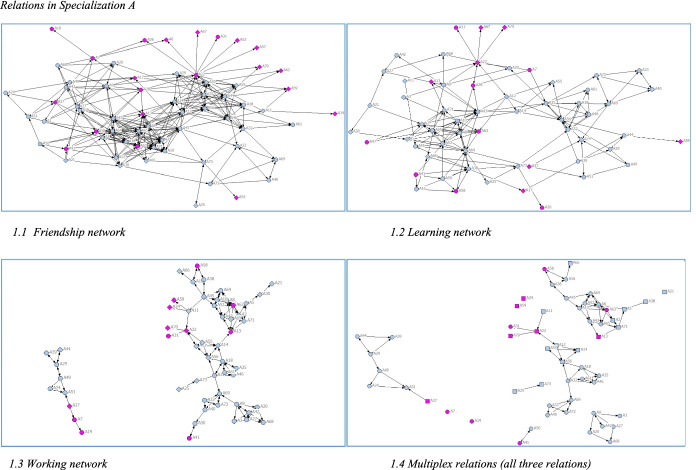
Relations in Specialization A

**Fig. 2:**
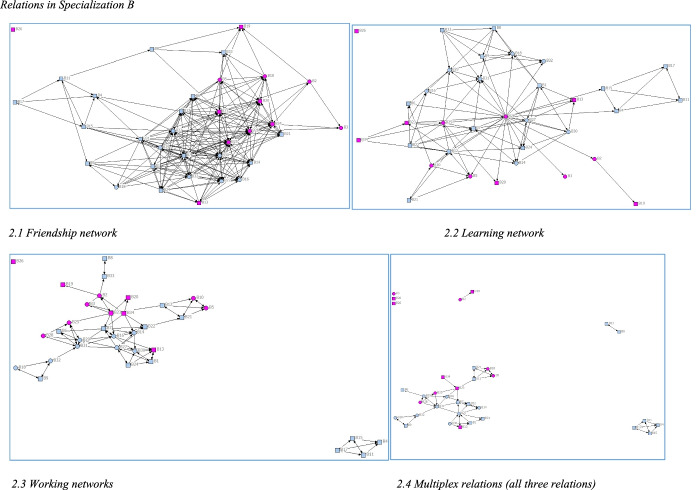
Relations in Specialization B

**Fig. 3:**
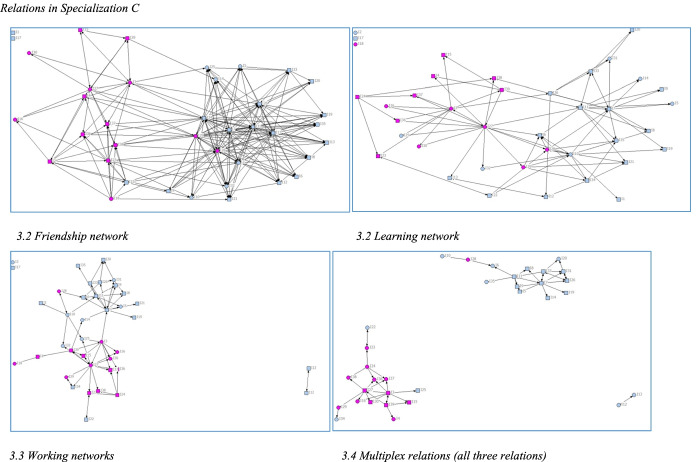
Relations in Specialization C

The different networks in each specialization are first presented as graphs, which are generated in NETDRAW (Figs. 1, 2, 3). These graphs are visual representations of the UCINET network data (friends with/learned from/work with), where each node is a student, and each line is a relationship between two students in the networks. The arrows indicate whether ties are reciprocal or unidirectional.

**Fig. 4:**
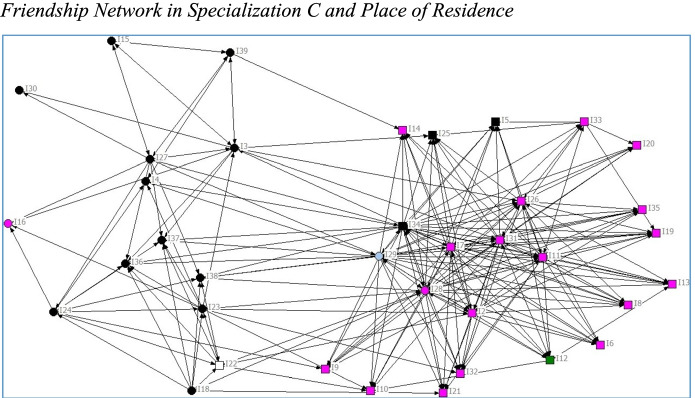
Friendship Network in Specialization C and Place of Residence. *Note:* Black nodes commuted along the southwest line, whereas white, blue, and green nodes commuted in other directions. Pink nodes were residents or commuted less than 30 min

Regarding the extent to which students develop relations with other students and the characteristics of networks formed (RQ1), Figs. 2, 4 indicate that there are differences between the specializations in overall structure, especially the friendship networks, where the network in Specialization C is denser and those in A and B less dense. In line with previous studies, students formed networks based on homophily*.* Native and immigrant students tended to cluster together in separate groups, as did students of the same gender. Educational background of parents and high school GPA had less impact on group formation. Place of residence had a greater impact, as commuter students partly cluster together depending on where they commute from. For example, Specialization C is divided into two clusters (Fig. 1). Commuter students living along the south-west train line form one cluster (black nodes) and non-commuters another (pink nodes). A large share of students who commute had an immigrant background (round nodes in Fig. 4).

The learning and working networks are less dense (Figs. 1, 2, 3), and students report having learned from and worked closely with a smaller number of students. In contrast, the working network (working networks in Figs. 1, 2, 3) are tightly grouped. Students form close working networks with a few students. Students they worked closely with were also marked as friends, and as a learning relation, indicating a strong prevalence of multiplex relations.

The descriptive statistics confirmed the visual analysis (Table 3). The friendship networks in all three specializations are the most connected, as they include 394, 316 and 250 ties, respectively. This confirms the picture of Specialization B as displaying a very dense friendship network (mean = 9.2) compared to A and C, which had a much looser structure (mean 6.4 and 5.3, respectively). The differences between specializations will be further discussed in the discussion section.

**Table 3:** Descriptive statistics of the networks

Specialization	Network	Total # of ties	Mean # of ties	Min # of ties	Max # of ties
A	Friendship	394	5.3	1	21
	Learning	181	2.5	1	11
	Working	154	2.1	1	9
B	Friendship	316	9.2	2	21
	Learning	124	3.6	1	24
	Working	109	3.2	1	7
C	Friendship	250	6.4	3	26
	Learning	94	2.4	1	14
	Working	77	1.9	1	12

Students in all three networks reported more learning relations than working relations. For example, in Specialization A, students on average had 2.5 learning relations but 2.1 academic work relations. Students in B reported having more learning than academic work ties; they had more relations overall than students in A and C, with 3.6 and 3.2 learning and academic work relations per node.

As could be discerned in Figs. 1, 2, 3, there was a tendency for students to form networks based on homophily, regarding gender and language background and place of residence. Krackhardt’s E-I index was calculated to measure the extent of homophily in the difference networks (Hanneman & Riddle, 2005).

Thus, the E-I indices confirmed the impression from Figs. 1, 2, 3 (Table 4) that students seemed to prefer forming networks with students who are similar to themselves, primarily regarding gender and language background. Interestingly, but perhaps not surprisingly, the tendency for homophily becomes stronger in the learning than in the larger friendship networks, and the strongest in the working networks.

**Table 4:** E-I index

Specialization	Attribute	Friendship	Learning	Working
A	Language background	− 0.478	− 0.571	− 0.597
	Gender	− 0.244	− 0.455	− 0.701
	Social background	− 0.115	− 0.135	− 0.130
	Place of residence	0.214	0.236	0.208
	Outcome	0.174	0.255	0.104
B	Language background	− 0,272	− 0,355	− 0.413
	Gender	− 0,076	− 0.065	− 0.138
	Social background	− 0,325	− 0.093	− 0.247
	Place of residence	0.177	0.274	0.175
	Outcome	0.387	0.241	0.107
C	Language background	− 0.376	− 0.511	− 0.662
	Gender	− 0.192	− 0.404	− 0.403
	Social background	0.232	− 0.132	0.095
	Place of residence	− 0.104	− 0.319	− 0.584
	Outcome	0.328	0.340	0.377

There is a strong tendency for homophily in Specialization C regarding Place of residence (Fig. 4). This means that students seemed to prefer working with students who live in the same place, and consequently who either do not commute or commute in the same direction. Thus, commuting strongly affected network formation. However, there seems to be an overlap between Place of residence and Language background in this sample, as a large share of the immigrant students commuted from the same larger metropolitan area.

QAP analyses were performed in UNICET to assess the associations between the different networks. The Jaccard coefficient, which is more suitable than the Pearson coefficient with binary relations (Hanneman & Riddle, 2005), indicates how similar two matrices are. In the case of Specialization B, for example, the Jaccard coefficient indicates (0.332) that if students are friends, there is a 33% probability that they also work together. If students work together, they are also likely to have learned from that person (0.465/47%).

Typically, the very core of each student’s network consisted of a few individuals with whom network members reported having all three relations, that is multiplex relations (Hanneman & Riddle, 2005). Relations were considered multiplex if nodes had all three possible relations (McCabe, 2016). This means that when a student works with another student, this is also someone the student is friends with and has learned from (see the Jaccard coefficients in Table 5). All coefficients are significant (*p* < 0.001), suggesting strong relationships that are unlikely to have occurred by chance (Hanneman & Riddle, 2005).

**Table 5:** Jaccard coefficients

Specialization	Friends/work	Friends/learn	Work/learn
A	0.369	0.373	0.450
B	0.332	0.310	0.465
C	0.258	0.269	0.527

All significant at *p* < 0.001

### Network Ties and Individual Performance

Table 6 displays descriptive statistics means, standard deviations, reliabilities, and zero-order correlations for variables, indicating a strong relation between Academic outcome and GPA, Language background and Work, Learning and Friendship Networks (*p* < 0.01). There is a less strong relation between Academic Outcome and Gender (*p* < 0.05). As expected, friendship, learning and work network size correlated significantly with academic outcome and with each other, however, VIF values between 2.2 and 2.8 and tolerance values between 0.36 and 0.46 indicate that there is no multicollinearity problem.

**Table 6:** Means, standard deviations, reliabilities and intercorrelations

	*Mean*	*SD*	1	2	3	4	5	6	7	8	9	10
1.Outcome	146.28	42.83										
2. GPA	16.91	1.99	.30^**^									
3. SweSAT	0.87	0.29	0.17	− 0.12								
4. Language background	0.63	0.48	.44^**^	0.14	.34^**^							
5 Social background	2.24	0.72	0.02	− 0.02	− 0.03	.20^*^						
6. Time commuting (hrs)	1.81	1.40	− 0.13	0.14	− .21^*^	− .24^**^	0.09					
7. Age	24.87	3.97	− 0.06	− .23^**^	.19^*^	.19^*^	− .21^*^	0.02				
8. Gender	0.56	0.50	.19^*^	.32^**^	− 0.09	0.08	− 0.15	0.04	− 0.00			
9. Work Ntw size	2.33	1.64	.45^**^	0.05	0.15	.39^**^	0.09	− 0.13	− 0.00	0.06		
10. Learning Ntw size	2.80	2.24	.48^**^	.23^**^	0.16	.42^**^	0.05	− 0.07	0.01	0.08	.74^**^	
11. Friendship Ntw size	6.63	4.54	.45^**^	0.11	0.14	.29^**^	0.11	− 0.11	− 0.06	− 0.00	.70^**^	.67^**^

Outcome in average credits achieved in nominal time. GPA is grade-point-average from Upper Secondary school; for gender: male = 0, female = 1; *p < .05; **p < .01; ***p < .001

RQ2 explored the relation between student friendship, learning and academic work network and academic outcome in this specific context (Table 6), without distinguishing between overlapping multiplex relations. Preliminary analyses indicated no violation of the assumption of multicollinearity. GPA, language background and gender were entered into Model 1, explaining 25% of the variance in academic outcome. Values for in-degree centrality of the friendship, learning and work networks were entered into Model 2 (Table 7). In Model 2, only GPA, language background and centrality in the Friendship network were significant, with language background recording a higher beta value (*beta* = 0.27, *p* < 0.001) than centrality in the Friendship network (*beta* = 0.22, *p* < 0.05) and GPA (*beta* = 0.19, *p* < 0.05). The total variance explained by Model 2 was 35%, *F* (5,143) = 13.94, *p* < 0.001, which means that the network variables explained an additional 11.6% of the variance in academic outcome, *R2* change = 0.116, *F* change (6, 143) = 8.548, *p* < 0.001. Hence, the analysis indicated that the number of socially focused friendship relationships in a student’s school-related network was positively related to that student’s performance, but this was not true of the work and learning networks.

**Table 7:** Predictors of academic outcome

	Predictors	*ß*	*F*	*df*	*R*^*2*^
Model 1	GPA	4.67**	16.65***	3, 143	.25
	Language backgr	36.13***			
	Gender	7.12			
Model 2	Friendship	2.09*	13.94***	6, 143	.35
	Learning	1.64			
	Working	2.74			
Model 3	Mplex	8.01**	16.78***	5, 143	.38
	Uplex	2.69**			

Outcome in average credits achieved in nominal time. GPA is grade-point-average from Upper Secondary school; for gender: male = 0, female = 1; *p < .05; **p < .01; ***p < .001

RQ 3 explored the relation between student multiplex relations and academic outcome. Due to multicollinearity issues, it was not possible to analyze the individual networks and the multiplex/uniplex networks in the same model. In Model 3 (Table 7), both centrality in the multiplex and that in the uniplex networks were significantly related to academic outcome. The multiplex network had a higher beta value (*beta* = 0.26, *p* < 0.01) than the uniplex network (*beta* = 0.20, *p* < 0.01). The total variance explained by Model 3 was 36%, *F* (5, 143) = 12.78, *p* < 0.001, which means that the network variables explained an additional 11.5% of the variance in academic outcome, *R2* change = 0.115, *F* change (5, 143) = 8.548, *p* < 0.001.

## Discussion

The relations between students’ study-related learning, working and friendship networks, as well as uniplex and multiplex networks, in a cohort of business students were explored using social network analysis. In contrast to previous research, the study was carried out at a teaching-intensive university where students follow the same program for 3 years and a large share of them commute, meaning that the context differs from that of previous SNA studies.

Previous research has offered inconclusive results concerning whether centrality in learning and work networks is linked to academic success (e.g., Rienties & Tempelaar, 2018; Tómas-Miquel et al., 2016). The present study confirms that centrality in the friendship network correlates significantly and positively with academic outcome, but this is not true of centrality in the working and learning networks. This is interesting, as the students themselves perceived that the work network was central to their academic success (Fjelkner-Pihl, 2021, Manuscript in preparation).

There was a substantial overlap between the friendship, learning and working relations. On average, students developed more friendship than learning and working relations, and slightly more learning than working relations. This is in line with previous studies in Anglo-Saxon countries (e.g., Rienties et al., 2015), but in contrast to Chen et al. (2012), who found that Chinese students reported fewer friendship and more academic ties, which they suggest may be partly explained by cultural factors. Students had more uniplex friendship ties than multiplex learning and work-related ones, in line with Shah et al.’s (2017) findings concerning a cohort of MBA students.

The substantial overlap between the three independent networks means that it is difficult to determine whether the effects of the friendship network can be solely attributed to the friendship itself or what part of the effect may be depend on the work-related component (Methot et al., 2016). Thus, student multiplex and uniplex ties were explored in relation to academic outcome. The multiplex relations had a higher beta value than did the uniplex relations, which indicates that the multiplex relations contributed substantially more to academic outcome than did the uniplex ties, although both correlated significantly and positively with academic outcome. This result supports the notion that the coexistence of friendship and instrumental ties in one relation creates a synergy, which may be both richer and more useful to students than uniplex relations only. This means that students have a network of a few trusted peers in the cohort and that this *significant network* is more important than social interactions with other individuals in the wider personal network, as previous research has pointed out in relation to university faculty peers (Becher & Trowler, 2001; Roxå & Mårtensson, 2015).

Given that student multiplex networks have been largely overlooked in the literature, it is time to acknowledge that students, just like academics, have several relations simultaneously. Arguably, these combined relations affect their performance, and these relations have also proved to be more resilient in the recent emergency transition to online teaching due to the Covid-19 pandemic. Therefore, it is important to provide students with opportunities to expand their relationships, so that rich links as well as multiplex relationships grow in number. As Felten and Lambert (2020) pointed out, it is in and around the physical and virtual classroom that students have the most opportunities to build relations with peers, and curriculum development, at least in Sweden, also needs to better acknowledge the social side of education and to provide these opportunities. How this can best be done differs depending on the context, as studies have indicated that commuter students, for example, rely on fewer relations than do campus students, which also left them more resilient when teaching transitioned to online platforms (REF).

One main contribution of the present study is to show how the tendency toward homophily increases the tighter the network gets, that is, the tendency is slightly more predominant in the learning and working relations, especially for gender and language background. This is problematic, mainly as higher education institutions risk cementing the homophily if students are not presented with ample opportunities to form relations with students of different backgrounds, thus denying them opportunities for both social and intellectual development which a more diverse network could potentially present them with. Academic success is important to integration in society and on the job market. Better integration in the program and better opportunities for all students, no matter their background, to work with and learn from a wider array of peers may offer more students both the affective and instrumental support they need to achieve academic success and social success.

The tendency for homophily based on place of residence in Specialization C, but not in the other two specializations, can be explained by the substantial overlap between language background and place of residence in this specific group. In this context, immigrant students mainly commuted from the same place, whereas native students commuted from other directions or lived on or close to campus. This is in line with previous research indicating that immigrant students are less prone both to move away from their families and to take student loans than are their native counterparts (London Higher, 2019; The Swedish Council for Higher Education, 2013). One may still wonder why this tendency is less prominent in the other two specializations. In Specialization B, the share of students with an immigrant background was lower and the entire group was better integrated (Fig. 2). Specialization A was less well integrated, and there is a clear pattern indicating that students from the same place tended to form relations with each other.

Finally, one important insight is how difficult it is to counteract homophily despite conscious efforts, on the part of program teachers, to mix students of different backgrounds in work groups during the first semester. The result indicated that there is an integration problem and intersectionality issue in the cohort, as commuter students with immigrant backgrounds and native Swedish students have separate networks. Immigrant students commuted longer distances and performed less well. It may be even more important to create opportunities that enable all students to form multiplex relations, that is, both affective and instrumental ties, that are not based on homophily. It has been argued that active measures to mix student and to help them form cross-cultural ties are important (Rienties & Nolan, 2014; Rienties et al., 2014). Despite the efforts to do so in this context, they did not seem to have any long-term effect, especially on the working and learning networks. Clearly, targeted efforts to create opportunity for students to meet and interact need to be extended beyond the first term if such efforts are to have a more long-lasting effect.

### Implications for Practice

Student multiplex networks have been largely overlooked in the literature. It is time to acknowledge that students, just like academics, have several relations simultaneously. These combined relations affect their performance, and this needs to be further explored. Further research should focus on students’ perception of their significant (multiplex) networks and on how they form and use these networks for academic support. This type of knowledge is important for teachers and curriculum planners, as it enables them to provide all students with opportunities to expand their social network, forming both uniplex and multiplex relations.

At the same time, the results reveal how difficult this can be. Despite teachers’ efforts to mix students as much as possible in group activities during their first year, the desired reduction in homophilic tendencies during network formation was not achieved. Interventions to support the evolution of multiplex friendship/learning/working relations are clearly difficult to plan or organize, something which has implications for diversity, equity, and inclusion in the program. These implications could be further explored in future research. One implication is to continue to acknowledge the importance of the self-selected work group and to strategically and systematically work with group activities in and around the classroom to enable all students to develop additional and sustainable working and learning relations, while at the same time acknowledging the importance of the friendship network as a vital source of inspiration and information. A more systematic work at the program level would perhaps enable more students to form both emotionally supportive and academically productive relations, which could lead to a better academic outcome for more students irrespective of background.

### Limitations

There are several limitations to this study, but they suggest paths for further research. First, the present study is based on self-reported relational data from 2016 and register data from 2018. The situation for today’s students is surely different from that of students in 2016, especially as much teaching has now transitioned online due to the current pandemic. At the same time, current research on student networks points to the importance of student relations (Felten & Lambert, 2020), and especially to overlapping, mulitplex relations, as these have been found to be more resilient than uniplex relations (Elmer et al., 2020; REF). The present article offers insights into students’ study-related social relations, and how these relations overlap to form multiplex work-focused networks. Future research could explore how students perceive these relations, and how they are formed and maintained, not only in a classroom situation, but also online.

Second, the networks were only constructed based on self-reported measurements at one point in time, thus offering a snapshot view of student friendship, learning and working relations, which may reduce the study’s validity. Students may have overstated or underestimated the number of relations in their networks. They may also have attempted to give “the correct answer” rather than their own perception of the number of relations in their network. All these biases may have led to a distortion of the data.

It must also be kept in mind that how students interpret the questions and what the different relations comprise may also differ. McCabe (2016), for example, left it to the students being interviewed to define friendship, and found great variability in the students’ conceptions. Furthermore, the labels given to the relations explored differ across studies, but the relations measured may be the same. For example, the working relations explored in one module—where teachers assign students, who mainly do not know each other, to teams (e.g., Rienties & Tempelaar, 2018)—may be different from the working relations explored in the present study, where students report on peers they mostly work with or have worked with during the entire course of their studies. Hence, it must be kept in mind that networks are fluid representations, snapshots of students’ perception of their friendship, learning and working relations, and must be treated as such.

At the same time, I feel the study offers a picture of how students perceive their social situation in this specific context. It reveals the complexity of the social relations in a cohort, how these relations overlap to form multiplex relations, and how these relations correlate with academic outcome. Future research could explore students’ multiplex relations in greater depth, looking at how these relations are formed and maintained, and how the academic culture fostered in these networks promotes or obstructs academic integration and success.

## Data Availability

Available upon request.

## Code availability

Available upon request.
